# Supplementary material for: A Likelihood Approach for Real-Time Calibration of Stochastic Compartmental Epidemic Models
Source: PLoS Comput Biol. 2017 Jan 17;13(1):e1005257. doi: 10.1371/journal.pcbi.1005257 (PMC5240920; doi:10.1371/journal.pcbi.1005257)
Supplement: S1 File — (TAR.GZ) [file pcbi.1005257.s014.tar.gz › HSPH_Online-SI-Revision/IPoi09/n10-extreme/n10-extreme_BENCH_table4.pdf]

1	R0	{2.68692, 12.5884}	{2.96307, 14.3219}	{{2.96307, 14.3219}, {2.96307, 14.3219}}	{10.2776, 13.7706}	inf	669.	762.919	{396., 1406.}	41.5859	0.148621	newly	306.	312.516	{162., 569.}	42.4942	0.171889	Reff	0.305503	0.252406	{0.208008, 0.300159}	25.386	ar	9089.	9382.58	{9331., 9427.}	3.23007
2	R0	{1.79802, 12.5412}	{2.85956, 19.8999}	{{2.85956, 19.8999}, {2.85956, 19.8999}}	{59.0394, 58.6762}	inf	841.	1417.95	{895., 2112.}	70.5756	0.209225	newly	397.	459.836	{280., 684.}	36.078	0.135974	Reff	0.656637	0.407459	{0.2708, 0.503283}	50.5479	ar	7412.	9302.69	{9253., 9345.}	25.5085
3	R0	{2.6174, 10.1105}	{2.92375, 10.9639}	{{2.92375, 10.9639}, {2.92375, 10.9639}}	{11.7044, 8.44073}	inf	459.	595.738	{295., 986.}	51.8554	0.165789	newly	256.	304.852	{156., 507.}	49.1803	0.171826	Reff	0.268022	0.24142	{0.204663, 0.297053}	18.0091	ar	9109.	9353.07	{9308., 9392.}	2.67939
4	R0	{2.27532, 14.3149}	{2.93488, 18.8445}	{{2.93488, 18.8445}, {2.93488, 18.8445}}	{28.9876, 31.6426}	inf	750.	1340.74	{770., 2461.}	81.0645	0.22575	newly	320.	449.115	{248., 854.}	50.6865	0.155409	Reff	0.473267	0.36788	{0.246236, 0.625423}	43.079	ar	8478.	9360.51	{9324., 9401.}	10.4094
5	R0	{2.96649, 18.0674}	{2.95376, 15.278}	{{2.95376, 15.278}, {2.95376, 15.278}}	{0.429127, 15.4389}	inf	1193.	554.484	{277., 1051.}	58.8297	0.412866	newly	386.	214.145	{105., 411.}	55.1521	0.354437	Reff	0.251262	0.230131	{0.198493, 0.270269}	14.8721	ar	9476.	9372.5	{9323., 9414.}	1.09223
6	R0	{2.94778, 16.0002}	{2.9627, 14.3601}	{{2.96307, 14.3219}, {2.96307, 14.3219}}	{0.506062, 10.2502}	inf	1090.	1086.93	{592., 1937.}	38.0914	0.16094	newly	362.	455.328	{226., 819.}	46.6489	0.155554	Reff	0.295957	0.297805	{0.22223, 0.395274}	24.7889	ar	9321.	9382.59	{9331., 9427.}	0.678707
7	R0	{2.17562, 11.7184}	{2.99939, 13.7076}	{{2.99939, 13.7076}, {2.99939, 13.7076}}	{37.8637, 16.975}	inf	793.	777.419	{438., 1191.}	35.2439	0.146648	newly	406.	335.774	{197., 503.}	40.7993	0.196909	Reff	0.496041	0.25147	{0.203059, 0.287941}	53.3703	ar	8345.	9410.45	{9366., 9453.}	12.7675
8	R0	{2.7904, 17.8966}	{2.96398, 17.7348}	{{2.96398, 17.7348}, {2.96398, 17.7348}}	{6.22061, 0.904082}	inf	1403.	941.339	{485., 1606.}	45.6384	0.264134	newly	475.	328.	{165., 577.}	46.275	0.262415	Reff	0.341266	0.278122	{0.214592, 0.362198}	29.5482	ar	9285.	9378.1	{9330., 9427.}	1.00266
9	R0	{2.51574, 16.376}	{2.96398, 17.7348}	{{2.96398, 17.7348}, {2.96398, 17.7348}}	{17.8174, 8.29751}	inf	1116.	1212.02	{656., 2013.}	34.5921	0.135964	newly	427.	425.613	{223., 693.}	35.227	0.150602	Reff	0.397235	0.324044	{0.232376, 0.443708}	34.6992	ar	8937.	9378.1	{9330., 9427.}	4.93562
10	R0	{2.56405, 18.1787}	{2.96398, 17.7348}	{{2.96398, 17.7348}, {2.96398, 17.7348}}	{15.5976, 2.44187}	inf	1437.	941.339	{485., 1606.}	46.314	0.271849	newly	467.	328.	{165., 577.}	45.7968	0.256942	Reff	0.441786	0.278122	{0.214592, 0.362198}	41.9966	ar	9005.	9378.1	{9330., 9427.}	4.14322
11	R0	{2.475, 17.1709}	{2.46477, 18.9032}	{{2.46477, 18.9032}, {2.46477, 18.9032}}	{0.413333, 10.0886}	inf	1270.	1161.77	{589., 1891.}	32.8268	0.16561	newly	447.	398.367	{197., 666.}	35.8315	0.180907	Reff	0.449708	0.507307	{0.336195, 0.678058}	32.6243	ar	8870.	8883.57	{8780., 8947.}	0.585494
12	R0	{2.14974, 11.8619}	{2.93488, 18.8445}	{{2.93488, 18.8445}, {2.93488, 18.8445}}	{36.5226, 58.8658}	inf	994.	1908.02	{1270., 2469.}	91.9534	0.270842	newly	494.	656.361	{416., 879.}	38.6341	0.134456	Reff	0.487776	0.557517	{0.304934, 1.13785}	46.8298	ar	8414.	9360.51	{9324., 9401.}	11.2492
13	R0	{2.20385, 14.4767}	{2.64373, 19.7855}	{{2.64373, 19.7855}, {2.64373, 19.7855}}	{19.9596, 36.6713}	inf	760.	1579.67	{970., 2338.}	107.851	0.29682	newly	319.	517.667	{318., 816.}	64.0195	0.19224	Reff	0.478235	0.533349	{0.347915, 0.805545}	35.2789	ar	8347.	9088.49	{9032., 9142.}	8.88334
14	R0	{2.35671, 13.8357}	{2.95376, 15.278}	{{2.95376, 15.278}, {2.95376, 15.278}}	{25.334, 10.4245}	inf	848.	776.435	{394., 1467.}	40.7125	0.185056	newly	361.	304.597	{160., 589.}	42.8335	0.209632	Reff	0.411717	0.254543	{0.211194, 0.321664}	39.4792	ar	8719.	9372.5	{9323., 9414.}	7.49513
15	R0	{1.91368, 10.1082}	{2.86656, 18.9813}	{{2.86656, 18.9813}, {2.86656, 18.9813}}	{49.7931, 87.7812}	inf	436.	1181.34	{661., 2303.}	170.951	0.3924	newly	261.	390.721	{215., 751.}	57.8921	0.16774	Reff	0.581759	0.349274	{0.251397, 0.520281}	47.9523	ar	7486.	9306.52	{9263., 9354.}	24.3191
16	R0	{2.48727, 14.882}	{2.86118, 15.5722}	{{2.86118, 15.5722}, {2.86118, 15.5722}}	{15.0329, 4.63782}	inf	1044.	1329.23	{805., 2241.}	38.3629	0.129887	newly	425.	525.721	{323., 900.}	38.3568	0.131142	Reff	0.402192	0.363389	{0.260081, 0.512151}	30.9039	ar	8894.	9309.66	{9255., 9350.}	4.67344
17	R0	{2.46795, 13.1915}	{2.99939, 13.7076}	{{2.99939, 13.7076}, {2.99939, 13.7076}}	{21.5337, 3.91237}	inf	950.	1085.48	{649., 1670.}	32.9338	0.121539	newly	457.	469.935	{256., 720.}	33.5498	0.136785	Reff	0.428189	0.300021	{0.224354, 0.360527}	42.8559	ar	8796.	9410.45	{9366., 9453.}	6.98558
18	R0	{2.96603, 16.7543}	{2.96947, 15.963}	{{2.96947, 15.963}, {2.96947, 15.963}}	{0.11598, 4.72297}	inf	1079.	1239.39	{728., 2087.}	36.2014	0.133125	newly	402.	477.871	{269., 825.}	39.8812	0.140131	Reff	0.284146	0.340445	{0.236667, 0.422259}	33.2491	ar	9394.	9383.39	{9320., 9450.}	0.407261
19	R0	{2.25216, 9.90465}	{2.99939, 13.7076}	{{2.99939, 13.7076}, {2.99939, 13.7076}}	{33.1784, 38.3956}	inf	643.	1085.48	{649., 1670.}	70.3306	0.202734	newly	379.	469.935	{256., 720.}	39.4842	0.133402	Reff	0.403812	0.300021	{0.224354, 0.360527}	40.0544	ar	8631.	9410.45	{9366., 9453.}	9.03084
20	R0	{2.70837, 18.1923}	{2.95349, 17.6633}	{{2.8833, 17.1847}, {2.96398, 17.7348}}	{9.05052, 2.9079}	inf	1399.	1601.56	{887., 2564.}	33.908	0.132798	newly	468.	569.147	{310., 953.}	39.208	0.145	Reff	0.365088	0.394665	{0.252827, 0.590425}	30.4912	ar	9183.	9379.51	{9329., 9438.}	2.1399
21	R0	{2.30288, 15.5877}	{2.79269, 19.984}	{{2.79269, 19.984}, {2.79269, 19.984}}	{21.2695, 28.2036}	inf	1029.	1312.57	{808., 1831.}	39.323	0.136153	newly	405.	417.8	{246., 593.}	31.0535	0.130016	Reff	0.441923	0.435441	{0.270612, 0.503522}	39.4687	ar	8674.	9238.12	{9175., 9301.}	6.50354
22	R0	{2.82208, 10.7544}	{2.91946, 11.8546}	{{2.91946, 11.8546}, {2.91946, 11.8546}}	{3.45065, 10.2302}	inf	586.	878.867	{491., 1242.}	56.2571	0.163355	newly	278.	422.933	{235., 583.}	58.8849	0.166315	Reff	0.244392	0.275101	{0.219251, 0.332818}	18.1598	ar	9333.	9349.67	{9293., 9392.}	0.3943
23	R0	{1.69923, 10.1366}	{2.93488, 18.8445}	{{2.93488, 18.8445}, {2.93488, 18.8445}}	{72.7182, 85.9055}	inf	558.	1340.74	{770., 2461.}	140.276	0.343457	newly	330.	449.115	{248., 854.}	47.839	0.149624	Reff	0.599318	0.36788	{0.246236, 0.625423}	50.0384	ar	7055.	9360.51	{9324., 9401.}	32.6791
24	R0	{2.64944, 19.054}	{2.85956, 19.8999}	{{2.85956, 19.8999}, {2.85956, 19.8999}}	{7.93073, 4.43949}	inf	1099.	1159.49	{705., 1852.}	35.5524	0.146039	newly	382.	367.459	{217., 547.}	36.3531	0.163161	Reff	0.35317	0.349874	{0.247066, 0.406915}	30.1089	ar	9147.	9302.69	{9253., 9345.}	1.70207
25	R0	{2.53191, 10.9214}	{2.8833, 17.1847}	{{2.8833, 17.1847}, {2.8833, 17.1847}}	{13.8785, 57.3489}	inf	571.	1476.77	{851., 2468.}	158.629	0.385158	newly	309.	539.475	{310., 895.}	76.6884	0.219254	Reff	0.310159	0.411702	{0.269012, 0.587905}	38.9317	ar	9009.	9313.56	{9263., 9361.}	3.38059
26	R0	{1.89155, 6.76944}	{2.9884, 12.8326}	{{2.9884, 12.8326}, {2.9884, 12.8326}}	{57.9868, 89.5666}	inf	234.	795.21	{425., 1339.}	239.833	0.485247	newly	188.	354.21	{193., 592.}	90.3826	0.235571	Reff	0.540416	0.248047	{0.205004, 0.284197}	54.7315	ar	7348.	9404.84	{9361., 9451.}	27.9918
27	R0	{2.63149, 15.6979}	{2.86118, 15.5722}	{{2.86118, 15.5722}, {2.86118, 15.5722}}	{8.72852, 0.800744}	inf	1228.	1329.23	{805., 2241.}	30.2224	0.118233	newly	463.	525.721	{323., 900.}	34.189	0.126446	Reff	0.367882	0.363389	{0.260081, 0.512151}	27.5596	ar	9104.	9309.66	{9255., 9350.}	2.25896
28	R0	{2.85141, 12.6992}	{2.92375, 10.9639}	{{2.92375, 10.9639}, {2.92375, 10.9639}}	{2.53699, 13.6646}	inf	776.	367.754	{172., 625.}	59.1199	0.416615	newly	347.	184.131	{83., 320.}	57.4526	0.385107	Reff	0.251494	0.218946	{0.193845, 0.253782}	15.7535	ar	9317.	9353.07	{9308., 9392.}	0.455716
29	R0	{2.54244, 12.9085}	{2.93488, 18.8445}	{{2.93488, 18.8445}, {2.93488, 18.8445}}	{15.4356, 45.9852}	inf	779.	1626.98	{994., 2550.}	108.855	0.296089	newly	343.	557.066	{317., 957.}	66.0613	0.196693	Reff	0.329754	0.447362	{0.269128, 0.831745}	46.0711	ar	9034.	9360.51	{9324., 9401.}	3.61422
30	R0	{2.10982, 11.7636}	{2.93488, 18.8445}	{{2.93488, 18.8445}, {2.93488, 18.8445}}	{39.1057, 60.1933}	inf	703.	1340.74	{770., 2461.}	91.8546	0.248327	newly	366.	449.115	{248., 854.}	41.2165	0.140723	Reff	0.486735	0.36788	{0.246236, 0.625423}	43.9283	ar	8224.	9360.51	{9324., 9401.}	13.8194
31	R0	{2.76312, 15.5396}	{2.96307, 14.3219}	{{2.96307, 14.3219}, {2.96307, 14.3219}}	{7.23638, 7.83611}	inf	1113.	1066.06	{592., 1912.}	37.1301	0.162576	newly	439.	447.242	{235., 819.}	39.2057	0.161154	Reff	0.324667	0.295103	{0.22223, 0.395274}	27.2653	ar	9220.	9382.58	{9331., 9427.}	1.76335
32	R0	{2.21727, 15.5439}	{2.42205, 19.4475}	{{2.42205, 19.4475}, {2.42205, 19.4475}}	{9.23568, 25.1134}	inf	936.	1315.74	{688., 1976.}	53.2217	0.183892	newly	399.	442.569	{244., 698.}	37.5205	0.157942	Reff	0.506203	0.606394	{0.365003, 0.870243}	40.12	ar	8359.	8811.98	{8743., 8883.}	5.4191
33	R0	{2.72912, 10.8159}	{2.99939, 13.7076}	{{2.99939, 13.7076}, {2.99939, 13.7076}}	{9.90319, 26.7356}	inf	395.	777.419	{438., 1191.}	97.0355	0.250031	newly	210.	335.774	{197., 503.}	64.2089	0.173578	Reff	0.252989	0.25147	{0.203059, 0.287941}	19.1682	ar	9196.	9410.45	{9366., 9453.}	2.33201
34	R0	{2.20928, 10.1308}	{2.92375, 10.9639}	{{2.92375, 10.9639}, {2.92375, 10.9639}}	{32.3395, 8.22344}	inf	446.	367.754	{172., 625.}	47.6402	0.240524	newly	238.	184.131	{83., 320.}	50.3788	0.266873	Reff	0.406066	0.218946	{0.193845, 0.253782}	46.0812	ar	8388.	9353.07	{9308., 9392.}	11.5053
35	R0	{2.10103, 15.2673}	{2.93488, 18.8445}	{{2.93488, 18.8445}, {2.93488, 18.8445}}	{39.6877, 23.4305}	inf	986.	1080.98	{581., 2054.}	39.6934	0.150979	newly	382.	357.803	{179., 691.}	41.5587	0.186047	Reff	0.508869	0.312305	{0.228921, 0.494234}	45.8508	ar	8264.	9360.51	{9324., 9401.}	13.2685
36	R0	{2.74914, 19.2775}	{2.79269, 19.984}	{{2.79269, 19.984}, {2.79269, 19.984}}	{1.58413, 3.66489}	inf	1441.	1312.57	{808., 1831.}	28.4918	0.135584	newly	436.	417.8	{246., 593.}	30.0459	0.135378	Reff	0.366735	0.435441	{0.270612, 0.503522}	36.9462	ar	9301.	9238.12	{9175., 9301.}	0.721249
37	R0	{2.78161, 16.0136}	{2.96398, 17.7348}	{{2.96398, 17.7348}, {2.96398, 17.7348}}	{6.55627, 10.7484}	inf	1033.	1516.56	{888., 2423.}	52.5263	0.166559	newly	388.	534.226	{299., 900.}	48.1211	0.156094	Reff	0.3146	0.39212	{0.254013, 0.581236}	36.2216	ar	9240.	9378.1	{9330., 9427.}	1.49455
38	R0	{2.4454, 8.33501}	{2.9884, 12.8326}	{{2.9884, 12.8326}, {2.9884, 12.8326}}	{22.205, 53.9602}	inf	243.	795.21	{425., 1339.}	227.247	0.468856	newly	147.	354.21	{193., 592.}	140.959	0.333256	Reff	0.306653	0.248047	{0.205004, 0.284197}	25.5721	ar	8853.	9404.84	{9361., 9451.}	6.23335
39	R0	{2.9154, 12.3861}	{2.9884, 12.8326}	{{2.9884, 12.8326}, {2.9884, 12.8326}}	{2.50394, 3.60485}	inf	596.	795.21	{425., 1339.}	46.9934	0.147988	newly	284.	354.21	{193., 592.}	42.8044	0.141066	Reff	0.225069	0.248047	{0.205004, 0.284197}	15.0124	ar	9371.	9404.84	{9361., 9451.}	0.417555
40	R0	{2.40923, 15.1963}	{2.96398, 17.7348}	{{2.96398, 17.7348}, {2.96398, 17.7348}}	{23.026, 16.7047}	inf	1025.	1516.56	{888., 2423.}	53.3895	0.16852	newly	421.	534.226	{299., 900.}	41.9431	0.145179	Reff	0.424988	0.39212	{0.254013, 0.581236}	36.4402	ar	8735.	9378.1	{9330., 9427.}	7.3623
41	R0	{2.63137, 15.238}	{2.93488, 18.8445}	{{2.93488, 18.8445}, {2.93488, 18.8445}}	{11.5343, 23.6678}	inf	1020.	1340.74	{770., 2461.}	43.2964	0.142022	newly	409.	449.115	{248., 854.}	37.5446	0.143644	Reff	0.336026	0.36788	{0.246236, 0.625423}	33.9675	ar	9150.	9360.51	{9324., 9401.}	2.30064
42	R0	{2.48769, 19.4593}	{2.46477, 18.9032}	{{2.46477, 18.9032}, {2.46477, 18.9032}}	{0.921337, 2.85776}	inf	1184.	1161.77	{589., 1891.}	33.6909	0.159271	newly	394.	398.367	{197., 666.}	37.1066	0.166602	Reff	0.413205	0.507307	{0.336195, 0.678058}	34.6103	ar	8932.	8883.57	{8780., 8947.}	0.635169
43	R0	{2.61199, 10.214}	{2.99458, 12.6606}	{{2.99519, 12.6436}, {2.99519, 12.6436}}	{14.6474, 23.9535}	inf	551.	1064.6	{568., 1639.}	94.6871	0.253661	newly	296.	494.935	{262., 736.}	72.6351	0.201577	Reff	0.299073	0.299356	{0.219349, 0.341751}	28.0011	ar	9061.	9409.32	{9360., 9461.}	3.8442
44	R0	{2.50994, 18.3382}	{2.64373, 19.7855}	{{2.64373, 19.7855}, {2.64373, 19.7855}}	{5.33041, 7.89227}	inf	1257.	1355.78	{761., 2255.}	34.1466	0.138857	newly	414.	441.667	{249., 763.}	36.5578	0.15007	Reff	0.47714	0.455683	{0.316719, 0.646392}	31.2252	ar	8827.	9088.49	{9032., 9142.}	2.96241
45	R0	{2.44283, 16.8338}	{2.93488, 18.8445}	{{2.93488, 18.8445}, {2.93488, 18.8445}}	{20.1426, 11.9444}	inf	1098.	1626.98	{994., 2550.}	52.6352	0.168608	newly	418.	557.066	{317., 957.}	43.2544	0.143736	Reff	0.427251	0.447362	{0.269128, 0.831745}	41.4803	ar	8878.	9360.51	{9324., 9401.}	5.43487
46	R0	{2.51815, 18.1462}	{2.79269, 19.984}	{{2.79269, 19.984}, {2.79269, 19.984}}	{10.9024, 10.1277}	inf	1305.	586.3	{271., 980.}	63.6143	0.455199	newly	464.	184.883	{80., 297.}	67.2234	0.512721	Reff	0.458051	0.280242	{0.224253, 0.290998}	44.9636	ar	8978.	9238.12	{9175., 9301.}	2.89727
47	R0	{2.88885, 16.8987}	{2.93488, 18.8445}	{{2.93488, 18.8445}, {2.93488, 18.8445}}	{1.59337, 11.5145}	inf	1290.	1626.98	{994., 2550.}	36.1571	0.127715	newly	468.	557.066	{317., 957.}	35.3125	0.12845	Reff	0.329618	0.447362	{0.269128, 0.831745}	46.0936	ar	9337.	9360.51	{9324., 9401.}	0.352555
48	R0	{2.58709, 13.8786}	{2.95376, 15.278}	{{2.95376, 15.278}, {2.95376, 15.278}}	{14.1731, 10.0831}	inf	1037.	1405.95	{831., 2388.}	44.1892	0.14349	newly	435.	562.065	{325., 954.}	41.5202	0.13689	Reff	0.330113	0.359382	{0.252842, 0.550876}	29.1047	ar	9146.	9372.5	{9323., 9414.}	2.47649
49	R0	{2.42959, 10.7132}	{2.99939, 13.7076}	{{2.99939, 13.7076}, {2.99939, 13.7076}}	{23.4525, 27.9506}	inf	676.	1085.48	{649., 1670.}	63.2086	0.1863	newly	359.	469.935	{256., 720.}	43.0856	0.139344	Reff	0.358122	0.300021	{0.224354, 0.360527}	34.3606	ar	8863.	9410.45	{9366., 9453.}	6.17682
50	R0	{2.60513, 13.2796}	{2.7499, 15.3124}	{{2.7499, 15.3124}, {2.7499, 15.3124}}	{5.55711, 15.3077}	inf	1051.	1465.98	{828., 2303.}	47.7778	0.156509	newly	465.	594.403	{328., 949.}	43.4305	0.151059	Reff	0.354037	0.43503	{0.297539, 0.620377}	32.8319	ar	9095.	9203.37	{9154., 9249.}	1.1919
